# Robust Building Extraction for High Spatial Resolution Remote Sensing Images with Self-Attention Network

**DOI:** 10.3390/s20247241

**Published:** 2020-12-17

**Authors:** Dengji Zhou, Guizhou Wang, Guojin He, Tengfei Long, Ranyu Yin, Zhaoming Zhang, Sibao Chen, Bin Luo

**Affiliations:** 1 Aerospace Information Research Institute, Chinese Academy of Sciences, Beijing 100094, China; zhoudengji19@mails.ucas.edu.cn (D.Z.); hegj@aircas.ac.cn (G.H.); longtf@aircas.ac.cn (T.L.); yinry@aircas.ac.cn (R.Y.); zhangzm@aircas.ac.cn (Z.Z.); 2 University of Chinese Academy of Sciences, Beijing 100049, China; 3 MOE Key Lab of Signal Processing and Intelligent Computing, School of Computer Science and Technology, Anhui University, Hefei 230601, China; sbchen@ahu.edu.cn (S.C.); luobin@ahu.edu.cn (B.L.)

**Keywords:** building extraction, high resolution image, semantic segmentation, deep learning

## Abstract

Building extraction from high spatial resolution remote sensing images is a hot spot in the field of remote sensing applications and computer vision. This paper presents a semantic segmentation model, which is a supervised method, named Pyramid Self-Attention Network (PISANet). Its structure is simple, because it contains only two parts: one is the backbone of the network, which is used to learn the local features (short distance context information around the pixel) of buildings from the image; the other part is the pyramid self-attention module, which is used to obtain the global features (long distance context information with other pixels in the image) and the comprehensive features (includes color, texture, geometric and high-level semantic feature) of the building. The network is an end-to-end approach. In the training stage, the input is the remote sensing image and corresponding label, and the output is probability map (the probability that each pixel is or is not building). In the prediction stage, the input is the remote sensing image, and the output is the extraction result of the building. The complexity of the network structure was reduced so that it is easy to implement. The proposed PISANet was tested on two datasets. The result shows that the overall accuracy reached 94.50 and 96.15%, the intersection-over-union reached 77.45 and 87.97%, and F1 index reached 87.27 and 93.55%, respectively. In experiments on different datasets, PISANet obtained high overall accuracy, low error rate and improved integrity of individual buildings.

## 1. Introduction and Related Work

Building extraction from high spatial resolution remote sensing images provides important information for urban-related applications such as smart cities, urban planning, population estimation and disaster management [1,2,3]. Since Liow et al. [4] began to study automatic building extraction from aerial remote sensing images in 1989, many well-known algorithms have been proposed. In high-resolution images, there are often large intra-class differences, while inter-class differences are relatively small. Additionally, remote sensing images are susceptible to differences in illumination, terrain, environment and atmosphere. These characteristics make it extremely difficult to exactly extract feature information from high-resolution remote sensing images. Buildings present large variability in types and shapes and tend to be smaller than other individual features such as roads and water bodies, increasing the difficulty of building extraction. Therefore, current algorithms for building extraction still need further research.

Traditional methods of building extraction are based on image segmentation or morphological operators. For example, Jin et al. extracted buildings from IKONOS images by constructing a differential morphological profile (DMP) with comprehensive structural, contextual and spectral information [5]. Bee et al. proposed an initialization algorithm of active contour model to improve the snake model for building extraction [6]. An algorithm based on morphology called bidimensional granulometry was developed by Sébastien et al., but this method is affected by objects of different types [7]. Another algorithm that uses morphology is the Morphological Building Index (MBI) [8], but it does not discriminate well features with similar spectral characteristics (e.g., open areas). Therefore, based on MBI, the Morphological Shadow Index (MSI) auxiliary building extraction was proposed [9]. There is also a semi-automated model that extracts buildings using interactive segmentation [10] through the steps of mean shift segmentation, region extraction and edge detection. These models and methods require artificial feature construction and features are often not universal for many other applications and datasets.

The accelerated spread of deep learning theory and remote sensing big data since the early 2010s [11] quickly promoted the development of information extraction-related algorithms [12], including for building extraction from high spatial resolution images. Many deep learning methods have been continuously presented for building extraction. Bittner et al. [13] and Shrestha et al. [14] put forward a fully connected network and conditional random field method for building extractions. Xu et al. [15] introduced the idea of ResNet to improve U-Net and used the guided filter to process the prediction results of the network. Although these networks may obtain higher accuracy, they all rely on post-processing for extracting buildings. With the development of deep learning models, many end-to-end training networks without pre-processing and post-processing have been put forward. Zhong and Huang et al. constructed end-to-end training networks based on fully convolutional network (FCN) and a deconvolution network (DeconvNet), respectively, for building extraction [16,17]. These methods have achieved good results on traditional image recognition tasks. However, there are various problems when they are applied directly to building extraction from high-resolution remote sensing images, and it becomes necessary to develop suitable models for this purpose. Yuan et al. [18] designed a convolutional neural network (CNN) capable of integrating multiple layers. Ji et al. [19] designed a CNN like U-Net and a feature pyramid network (FPN), and both achieved good results on a public dataset. Yang et al. compared four methods of branch-out CNN, FCN, conditional random fields as recurrent neural networks (CRFasRNN) and SegNet for large-scale mapping of buildings in the United States [20]. Although these networks use multiple convolution layers to expand the scale of the receptive field, these features still obtain local context information.

Non-local networks provide a new solution for global feature extractions related to buildings [21]. The principle of non-local networks is to use the self-attention mechanism for obtaining the dependency relationship between any two pixels in the feature map and derive long-distance dependency relationships. Based on this concept, semantic segmentation networks based on self-attention mechanisms have developed rapidly. Fu et al. designed a position attention module and a channel attention module using the self-attention mechanism to capture the position and channel dependencies [22]. Experiments have shown that a state-of-the-art effect is achieved on current mainstream datasets. The self-attention mechanism in [23] calculates feature maps in a way that maximizes expectations and achieves a current optimal effect. Simultaneously, the semantic segmentation networks of self-attention mechanism have been shown to be able to extract global features better [24,25,26].

In addition, with the development of deep learning technologies [27], the structure of a deep learning network becomes increasingly complex and needs more samples [28]. The deep learning networks for building extraction often have complex structure and tedious steps. For example, Liu et al. [29] used multiple spatial residual inception (SRI) structures and crisscrossed feature map connections for building extraction. Sun et al. [3] and Huang et al. [17] proposed a method that trained multiple existing deep learning models and fused the outputs to obtain the extraction results, which required several times more work than other end-to-end methods. Li et al. [30] used generative adversarial networks for building extraction. In summary, the above models have either a complicated model structure or are difficult to train with parameters.

High-resolution remote sensing images have rich spectral, textural and spatial context features. To make full use of the overall characteristics of buildings, the long-distance dependencies for global context information is needs to be combined with local features for building extraction. It is necessary to design a simple network that can integrate local and global features. Inspired by two models [22,31], we propose a novel network called Pyramid Self-Attention Network (PISANet). The structure of the PISANet is mainly composed of the backbone and PISA module. We validated PISANet with public building datasets and compared its performance with that of other widespread networks, such as SegNet, PSPNet and DeepLab V3. Experimental results have demonstrated that the PISANet can make full use of the local and global information together, maintain the integrity of the building shape and greatly reduce the occurrence of background area error.

## 2. Methods

### 2.1. Motivation

In traditional CNN, the network captures a wider range of contextual information by increasing depth of network. However, too large network depth will have some side effects, such as gradient disappearance and gradient explosion [32]. The residual network (ResNet) partly overcomes the problem in the training of deep network, since it uses skip connection in different layers to stabilize the training. However, for building extraction with semantic segmentation, there is still a contradiction between global context information and local context information. The local context information is mainly the feature closely around the pixel, which is helpful to recognize the details. Global context information is the dependency of any pixel in the whole picture and can distinguish confusing semantic information. In ResNet, the connections between layers are mainly at the same scale, which is hard to avoid the contradictions between global and local features. 

The self-attention mechanism can establish the connection between different scale layers. This special inter-scale connection can coordinate the relationship between different scales and levels, and then the contradictions between the global and the local will be alleviated in semantic segmentation. Therefore, this paper designs a network PISANet with portable structure, which can coordinate local and global context information well to improve the results of building extraction. In the following, the structure of PISANet will be presented in detail.

### 2.2. PISANet

**Figure 1:**
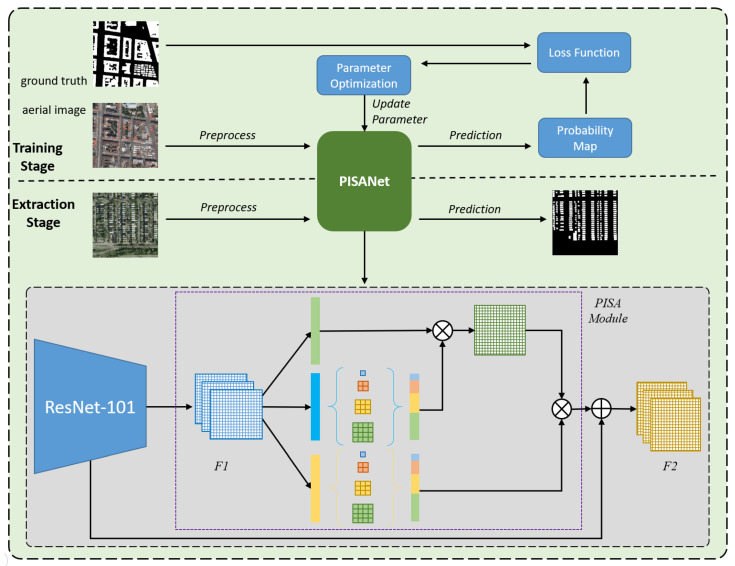
The flowchart of the proposed method and structure of the Pyramid Self-Attention Network (PISANet). The flowchart is divided into training stage and extraction stage. In the training stage, the input is a remote sensing image and corresponding label, and the output is a probability map. In the extraction stage, the input is a remote sensing image, and the output is the extraction result of the building.

The structure of PISANet (Figure 1) is mainly composed of the backbone and PISA module. The backbone can be any feature extraction network, such as VGG or ResNet. Because ResNet [32] has an excellent feature extraction ability, ResNet-101 was adopted as the backbone in the proposed PISANet. The feature map (it is a two-dimensional array with a large number of channels. Generally speaking, there are 64 to 2048 channels. In particular, we can also regard the red, green and blue (RGB) bands of aerial imagery as a 3-channel feature map) F1 from the backbone network was then sent to the PISA module. The PISA module was used to generate the global feature map F2, which contains the long-distance context learned from the training dataset. The local representation feature map F1 and the dense context global feature map F2 were combined and sent into the segmentation layer to generate the probability map (a 2-channel feature map, the first channel is the probability that each pixel is classified as a non-building, and the second channel is the probability that each pixel is classified as a building).

#### 2.2.1. Backbone Network

With strong learning ability, ResNets can dramatically alleviate the difficulty of training deep network. There are five kinds of ResNets, which are Resnet-18, Resnet-34, Resnet-50, Resnet-101 and Resnet-152. Due to the tradeoff between the computation and performance, this paper use ResNet-101 as the backbone of PISANet.

**Figure 2:**
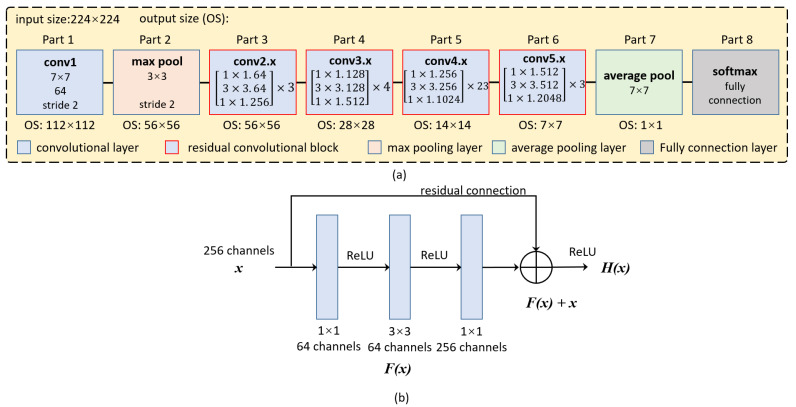
(**a**) The structure of ResNet-101, it can be divided into 8 parts. (**b**) One of the residuals learning structures for the first residuals block of ResNet-101.

Figure 2a is the network structure diagram of ResNet-101, which can be divided into 8 parts, including 5 structures. The first part is a convolution layer. The convolution kernel size of the input image is 7 × 7, and the stride is 2. In the process of learning features, the size of the feature map is halved from the input size 224 × 224 to the output size 112 × 112. The second part is the maximum pooling layer. The maximum value within the range of 3 × 3 pixels is taken as the output, and the size of the feature map is further halved to reduce the output size to 56 × 56. The third to sixth parts are residual convolution blocks, which are also the core of ResNet. This part mainly increases the depth of the network for further residual learning. The first residual convolution block does not change the size of the output feature map, and the size of the output feature map for the other three blocks is halved. The seventh part is the average pooling layer. The average value within the range of 7 × 7 pixels is taken as the output. The eighth part is the full connection layer composed of 1000 neurons, corresponding to 1000 categories. The output is the probability of the input image for the 1000 categories. The category with the highest probability is the final output class.

Figure 2b is a residual learning structure in the first residue convolution block of ResNet-101. Residuals learning means that if the input feature map is x and the output feature map is H(x), then the expected residuals are F(x) = H(x) − x. Under extreme condition, if the learned residual F(x) = 0, then H(x) = x, which ensures that the input feature x will not be lost and the performance of the network will not decrease. In practice, the residual will not be 0, which means that several convolutional layers in the residual learning structure will learn new features on the basis of the input feature map to improve the performance of the network. In each residual structure, it consists of three convolutional layers, which are 1 × 1 convolutional layer, 3 × 3 convolutional layer and 1 × 1 convolutional layer. As in Figure 2b, the first 1 × 1 convolutional layer is for dimensionality reduction, compressing 256 channels to 64 channels; the second 3 × 3 convolutional layer is to further learn features at higher levels, where the number of channels in the output feature map is 64 channels; the third 1 × 1 convolution is a dimension increase, which increases the 64 channels to 256 channels. There are 3 residual structures in the first residual convolution module in ResNet-101, that is, the 3 residual learning structure are connected end to end. Similarly, the second to fourth residual convolution blocks are with the same structures, but the number of channels is different. The residual connection is similar to a short circuit, so it is also called a shortcut connection.

#### 2.2.2. Modifications Based on Backbone

Since ResNet-101 is a network used for image classification, it needs improvements to be applied to pixel-level classification. First, we remove the 7th and 8th parts in ResNet-101. These two parts have no effect on our task and will also lose information. In the 5th and 6th part, the size of the output feature map is halved, because it contains a down-sampling operation, that is, the convolution kernel performs convolution with stride of 2. We remove the down-sampling operation in these two parts and replace it with dilated convolution, so that the size of output feature map is changed to 28 × 28. The outcome of this design is the reduction in the size of the output feature map obtained through the backbone (1/8 of the size of the input), while retaining more spatial information without adding additional parameters.

#### 2.2.3. Self-Attention

The convolutional layer in the convolutional neural network limits the range of the local receptive field (a pixel on the feature map corresponds to the range of pixels on the input) so that only short-distance context information can be obtained. Global context information has an important impact on network performances. In 2017, Ashish et al. [33] proposed to use the non-local module to obtain global features, which were later introduced into computer vision.

**Figure 3:**
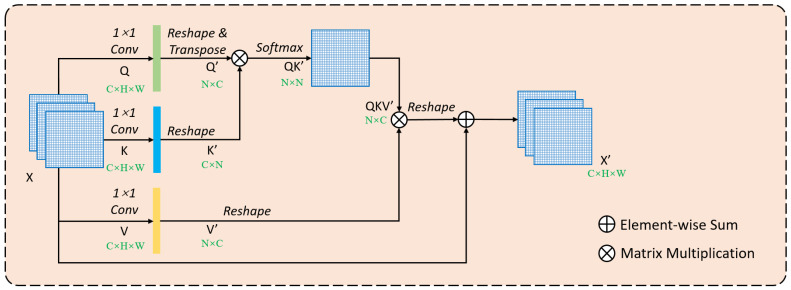
The structure of self-attention [22].

The non-local network significantly increases the visual range and can obtain global context information from getting pairwise dependencies of all pixels (Figure 3). The input feature map X enters each of the Q, K and V branches and performs a 1 × 1 convolution in each branch to achieve a dimensionality reduction. After the feature map X enters the Q branch convolution, its size is C × H × W (C represents the number of channels in the feature map, H represents the height of the feature map and W represents the width), which is then reshaped to C × N. After transposing, a feature map Q’ of size N × C is obtained, where N = H × W. In branch K, the convolution changes the size to be K prime of C × N, and the convolution changes the size to be V prime of N × C. After X enters the K and V branches, the feature maps K’ and V’ of C × N and N × C sizes are obtained, respectively. At this time, the feature maps Q’ and K’ in the Q and K branches are matrix-multiplied to obtain QK’. The purpose of this step is to obtain the relationship between two pixels in the input feature map. The N × N weight information is obtained by normalizing QK’ through SoftMax (a function that normalizes a weight value to a range of 0~1), which calculates the influence of each pixel in all K’ on each pixel in Q’, the output is called the attention map. Then, QK’ and V’ are matrix multiplied to obtain a feature map QKV’ with a size of N × C. After resizing, it is added to the input feature map X to obtain a feature map X’ with a size of C × H × W. However, the non-local module consumes a lot of computing resources and graphics processing unit (GPU) memory in the actual processing. Assuming that the input feature map size is C × H × W, the computational complexity of similarity relationship between any two pixels (to represent the time and space required by the algorithm, the higher the value means the more complex it is) is O (CN^2^ = CH^2^W^2^) in the Q and K branches. When calculating the influence of all positions on a certain pixel, a matrix multiplication operation with a complexity of O (CN^2^ = CH^2^W^2^) is still required. The non-local block consumes a lot of computing resources to generate a huge attention map, while acquiring global context information.

#### 2.2.4. Pyramid Self-Attention

**Figure 4:**
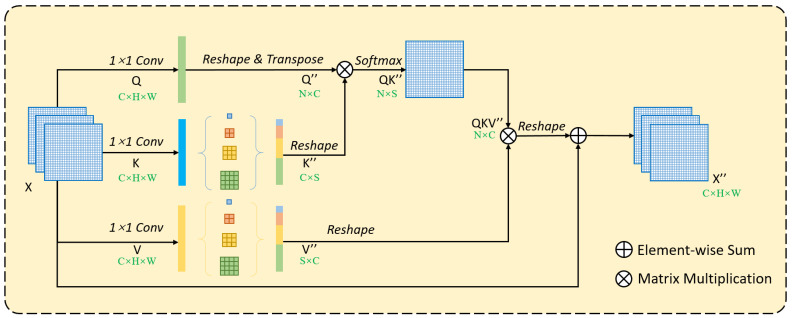
The structure of PISA module.

To retain spatial information as much as possible and effectively reduce the amount of calculation and GPU memory usage, the key is to reduce the size of the attention map generated in non-local, while obtaining dense context information. This can be achieved by simply changing the number of pixels in each channel in the K and V branches [31]. As shown in Figure 4, the size of the feature map Q” obtained in the Q branch is N × C, and the size of the feature map K” obtained in the K branch is C × S. After matrix multiplication of Q” and K”, the feature map QK” with size N × S is obtained, and the size of feature map QKV” after matrix multiplication of QK” and V” is N × C, which is the same as QKV’ in Figure 3. At this time, as long as the condition of S << N is satisfied, the computational complexity can be reduced to O(CNS), which is significantly lower than the complexity O(CN^2^) of the original self-attention. The calculation includes the following two processes:

(1) The calculation process of Self-Attention (Figure 3):

(R^N × C^ × R^C × N^) → R^N × N^ × R^N × C^ → R^N × C^ (1)

(2) The calculation process of Pyramid Self-Attention (Figure 4):

(R^N × C^ × R^C × S^) → R^N × S^ × R^S × C^ → R^N × C^ (2)

**Figure 5:**
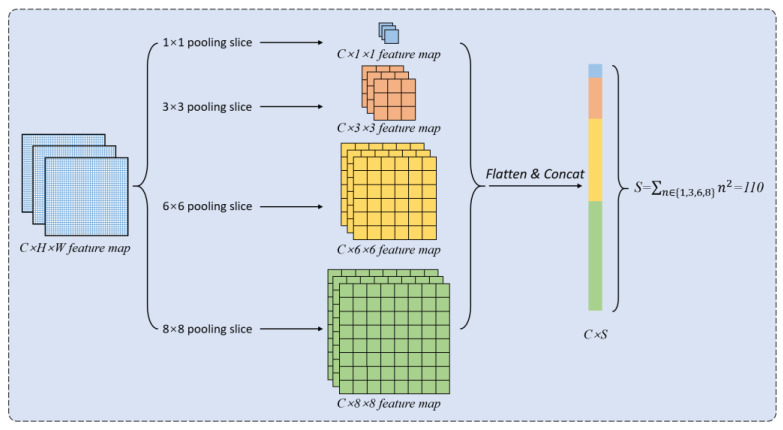
Schematic diagram of pyramid adaptive pooling [34].

To reduce complexity while preserving spatial information as much as possible, we introduced the Spatial Pyramid Pooling structure [31,34], which adopts multiple pooling kernels of different sizes for adaptive pooling (Figure 5). The feature map of C × H × W size is entered into SPP, and the feature maps of C × 1 × 1, C × 3 × 3, C × 6 × 6 and C × 8 × 8 are obtained after adaptive pooling. The four feature maps are split together after pixel decomposition, and a feature vector of C × S length is obtained. *S* is equal to the number of pixels in each channel in the four feature maps:

$$S=\underset{n\in \left\{1,3,6,8\right\}}{\sum }n^{2}=110$$

where *n* represents the width or height of each feature map after pooling. It can be seen that *S* << N (assuming that the feature map size is 32 × 32, then N = 32 × 32 = 1024, *S* = 110 << N), the complexity is also reduced to O (CNS).

### 2.3. Preprocessing

In order to learn more effectively, accelerate model fitting and improve the training accuracy, the dataset needs to be pre-processed before it is fed to the network. Two preprocessing operations are required before starting the deep learning experiments. The first step is to normalize the input image band value and subtract the average value of the RGB three bands. The purpose of image normalization is to move the image pixel distribution to the center position for obtaining stable weights and variances and improve training efficiency. The second step is to augment the dataset. Because samples in the training dataset are limited, the sample diversity needs to be increased by rotation, mirroring, random noise and blurring. This improves the robustness of the model and prevents it from overfitting.

### 2.4. Training Strategy

The process of building extraction in this paper was divided into training and prediction (Figure 1). In the training part, the image and the corresponding label were used by the network for training after preprocessing. In each training epoch, according to the network parameters learned, the corresponding output probability map was obtained for the input image. The output probability map and the ground truth label used the loss function to calculate the gap between the current model and the expected model. Then, the parameters were optimized and updated through backward propagation to complete an epoch of training. When the model training was completed, it entered the extraction stage, in which the model output the predicted building extraction effect map from the test set image. The extraction accuracy of the model was assessed by comparing the prediction with the ground truth.

In this paper, the batch input increased the model stability. The batch size parameter (the number of samples entered at the same time during each training iteration) was set to 5, the initial learning rate (represents the speed at which information accumulates in the neural network over time) was set to 0.01 and the aerial image and the corresponding ground truth are cropped to a size of 400 × 400; the learning rate was dynamically updated with the epoch of training.

### 2.5. Advantages and Disadvantages

In this paper, the proposed PISANet combine local context information and global context information to improve the feature expression ability of buildings extraction. With this comprehensive feature representation, some interference targets similar to buildings (such as containers) can be correctly distinguished by PISANet without being misclassified. The integrity of the building can also be well guaranteed, and the phenomenon of missing is reduced (Section 4 experimental part). In terms of network structure, PISANet only contains two modules, the structure is simple and easy to implement, and there is no intricate structure. Some disadvantages in similar types of networks still existed in PISANet. For some too small buildings, all of them cannot extract their very tiny details.

## 3. Dataset and Evaluate Indicators

### 3.1. Dataset

To evaluate the proposed method, two public building datasets, the Inria Aerial Image Labeling Dataset [35] and the Wuhan University (WHU) Building Dataset [36], were used for PISANet validation. These two datasets cover a wide range of building types, different shapes, and are both at high spatial resolution.

The Inria Aerial Image Labeling Dataset, released by Maggiori et al., covers an area of 810 square kilometers, with a total of 360 pictures, each with 5000 × 5000 pixels. The dataset includes ten densely populated cities and remote villages (Austin, Bellingham, Bloomington, Chicago, Innsbruck, Kitsap, San Francisco, Western and Eastern Tyrol and Vienna). The spatial resolution of the images reaches 0.3 m and the shape and structure of the buildings are clearly visible. The dataset is divided into a training set and a test set. Only the training set is completely public. For training and prediction, the 180 training samples are divided into 160 training subsets, 10 verification subsets, and 10 test subsets. A special characteristic of this dataset is that different cities are contained in different subsets; for example, Austin is part of the training set and Bellingham belongs to the test set.

The WHU Building Dataset is a building dataset containing aerial imagery and satellite imagery released by Wuhan University. In this paper, a subset of aerial imagery from this dataset was selected. It contains 8188 non-overlapping 512 × 512 pixels pictures with a spatial resolution of 0.075 to 0.3 m, covering 450 square kilometers of Christchurch in New Zealand. The dataset is divided into a training set (4736 sheets, containing 130,500 buildings), a validation set (containing 1036 sheets, 14,500 buildings) and a test set (2416 sheets, containing 42,000 buildings).

Table 1 shows some characteristics of the Inria Aerial Image Labeling Dataset and WHU Building Dataset. It is a pity that the official websites of these two datasets did not publish the relevant parameters of aerial imagery, such as flight altitude, sensors, etc. Therefore, we only list some characteristics of the dataset itself.

Because of GPU memory limitations, the raw size of a single training sample was too large (the Inria Aerial Image Labeling Dataset consists of 5000 × 5000 pixels per image). We randomly cropped the input training sample to 400 × 400 pixels size and performed training after data augmentation.

### 3.2. Evaluate Indicators

To facilitate comparison and evaluation, the following five widely used evaluation indicators were selected for model evaluation: overall accuracy (OA), intersection over union (IoU), precision rate, recall rate and F1 score. The specific formulas are as follows [14]:

(3) $$\mathrm{OA}=\frac{\mathrm{TP}+\mathrm{TN}}{\mathrm{TP}+\mathrm{TN}+\mathrm{FP}+\mathrm{FN}}$$

(4) $$\mathrm{IoU}=\frac{\mathrm{TP}}{\mathrm{TP}+\mathrm{FP}+\mathrm{FN}}$$

(5) $$\mathrm{precision}=\frac{\mathrm{TP}}{\mathrm{TP}+\mathrm{FP}}$$

(6) $$\mathrm{recall}=\frac{\mathrm{TP}}{\mathrm{TP}+\mathrm{FN}}$$

(7) $$F1=2\times \frac{\mathrm{precision}\times \mathrm{recall}}{\mathrm{precision}+\mathrm{recall}}$$

where TP is true positive, TN is true negative, FP is false positive and FN is false negative.

## 4. Experiment and Discussion

To verify the effectiveness of PISANet, classic networks such as SegNet [37], PSPNet [38] and DeepLab V3 [39] were used for comparison. Two experiments were analyzed and discussed in detail as follows.

### 4.1. Inria Aerial Image Labeling Dataset Experiment

**Figure 6:**
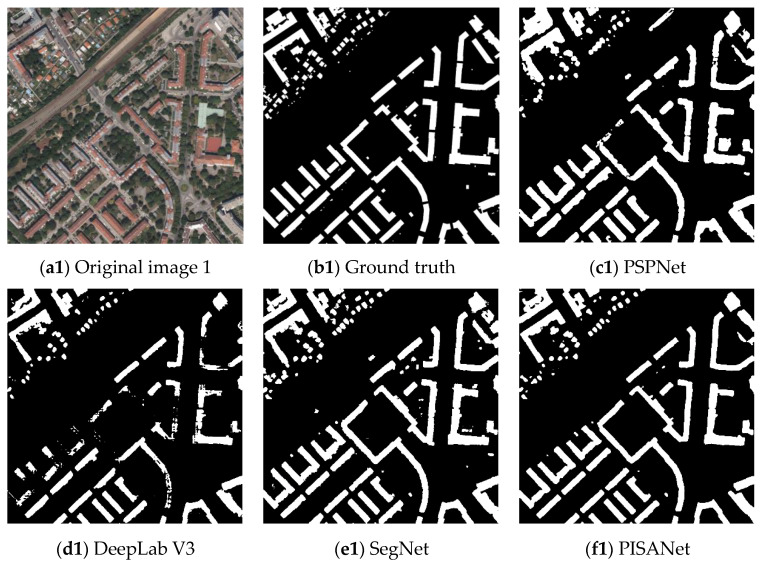

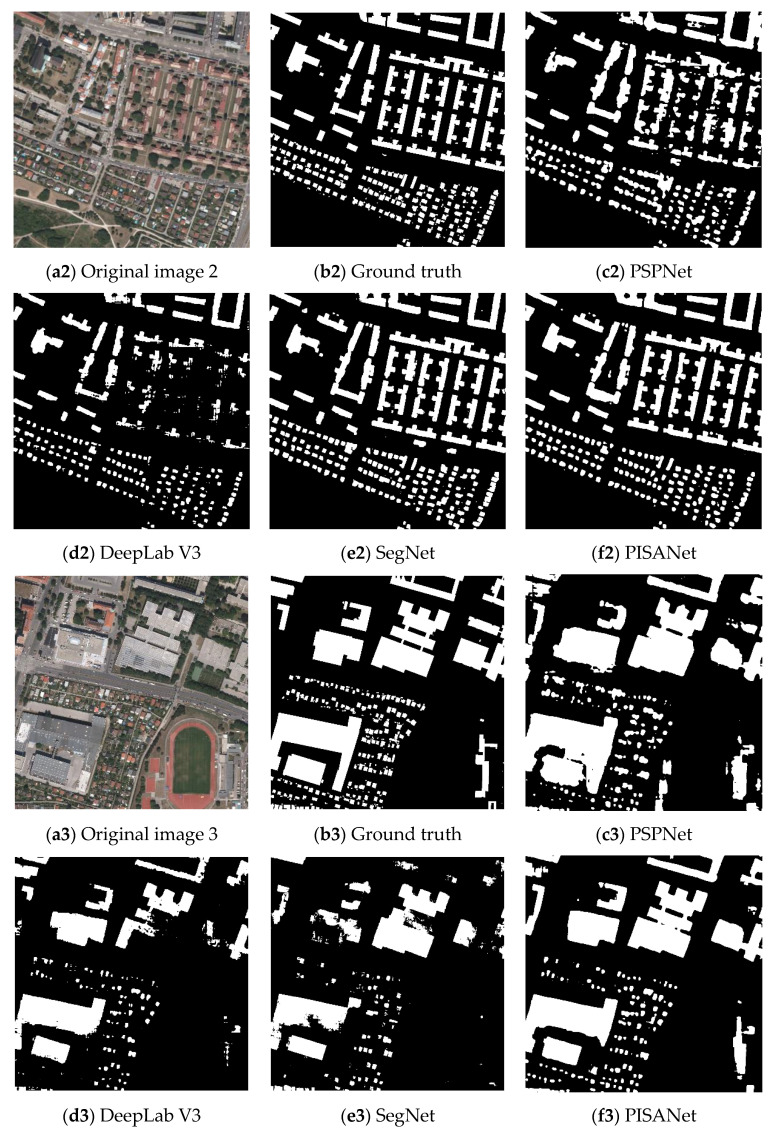

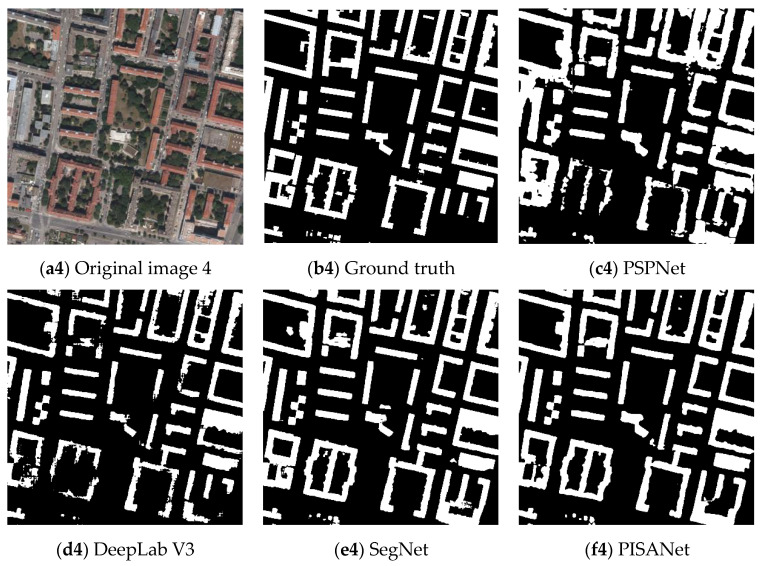
Visual comparison of different methods on the Inria Aerial Image Labeling Dataset.

A qualitative assessment of the various methods on the Inria Aerial Image Labeling Dataset can be seen in Figure 6. We selected four representative areas in the test set to display. The original image contains the different shapes of large buildings and small buildings. From the extraction results, we can observe that PSPNet and DeepLab V3 cannot distinguish the building and the ground where they are similar. SegNet and PISANet have a slight advantage. Overall, SegNet and PISANet can handle large buildings, small buildings, and special-shaped buildings with ease. Although SegNet performed well, the integrity of the building was not maintained. This is because SegNet is a network based on an encoding–decoding structure, and the convolution kernel is mainly used to extract features in an image. The convolution kernel obtains short-distance context information due to the limitation of the receptive field, where the PISA module uses the non-local module to capture global information. Therefore, the overall characteristics of the building can be obtained, and the building can be more completely extracted, which can be seen more clearly in the extraction of large buildings. In the building extracted by PISANet, there are few voids inside a single building, reflecting the advantages of the PISA module.

Table 2 lists the experimental results of PISANet and the three comparison methods on the Inria Aerial Image Labeling Dataset. It is shown that PISANet has obvious advantages in OA, IoU and F1 indicators. Although precision and recall are not both the highest, it is not realistic to expect both precision and recall to have the best values. The F1 indicator integrates both precision and recall and thus is a more preferred metric. OA is a combination of the proportion of the number of pixels that are completely predicted correctly in the two areas with building and without building. The IoU indicator is the total number of pixels that are actually predicted in the building category to the total number of pixels of all building categories (whether it is a predicted label or a ground truth label). Based on the above experiments, PISANet surpasses the other model in these three indicators, indicating that PISANet has certain advantages in building extraction.

**Figure 7:**
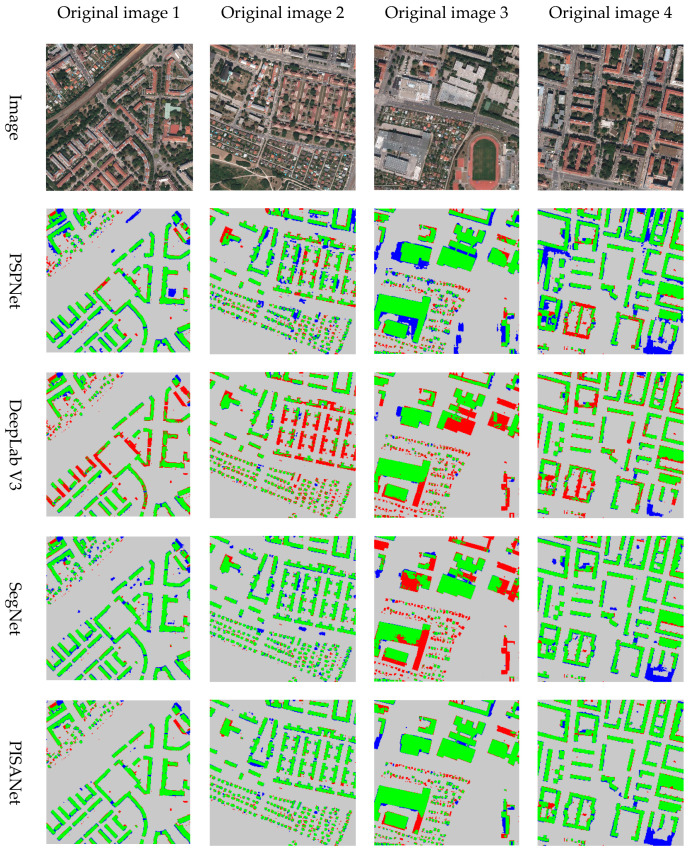
Visualization of indicators of different models on the Inria Aerial Image Labeling Dataset, where gray is TN, green is TP, red is FN, blue is FP.

In order to qualitatively show the accuracy of the results, the predicted results are displayed in color (Figure 7). The green part is true positive, the gray part is true negative, the blue part is false positive and the red part is false negative. When the extraction performs well, the green (true positive) and gray (true negative) are the majority, but the blue (false positive) and red (false negative) are minority. Red (false negative) indicates missing building information, and blue (false positive) indicates incorrect building information. From Figure 7, it can see that red and blue are less in PISANet than in the results of the other three methods, indicating a better performance. PISANet is also slightly better than the other methods at discriminating non-buildings (the blue part is less). Due to certain errors in the ground truth, some buildings are represented by non-buildings on the ground-truth label, but the predicted result is considered to be buildings, which leads to more blue areas (the building in the lower right corner of original image 4 is a non-building in the ground truth label).

### 4.2. WHU Building Dataset Experiment

**Figure 8:**
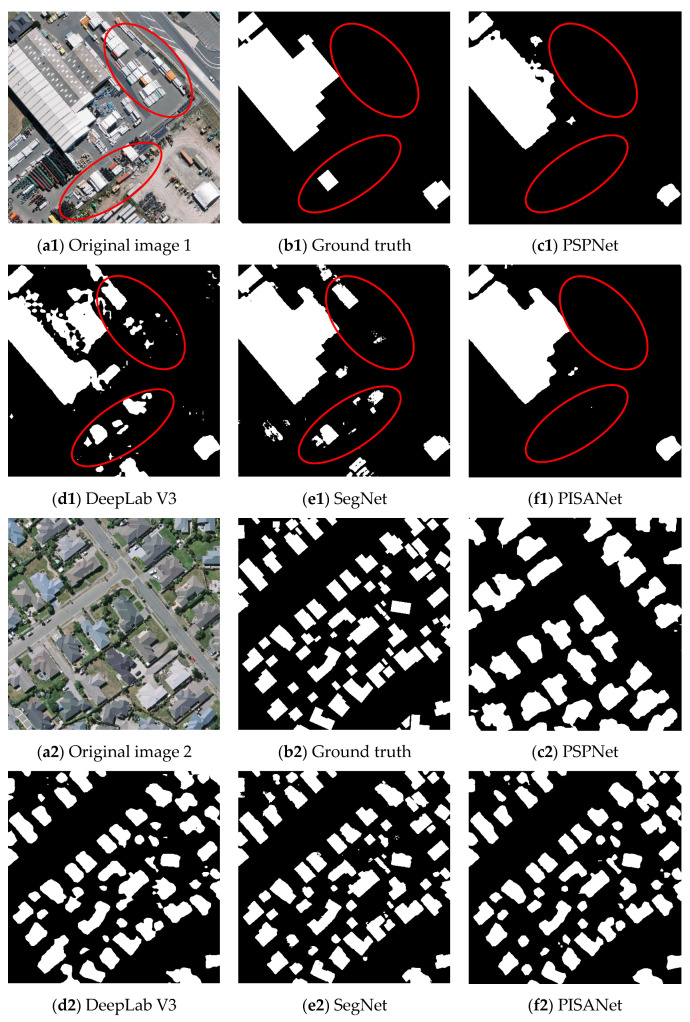

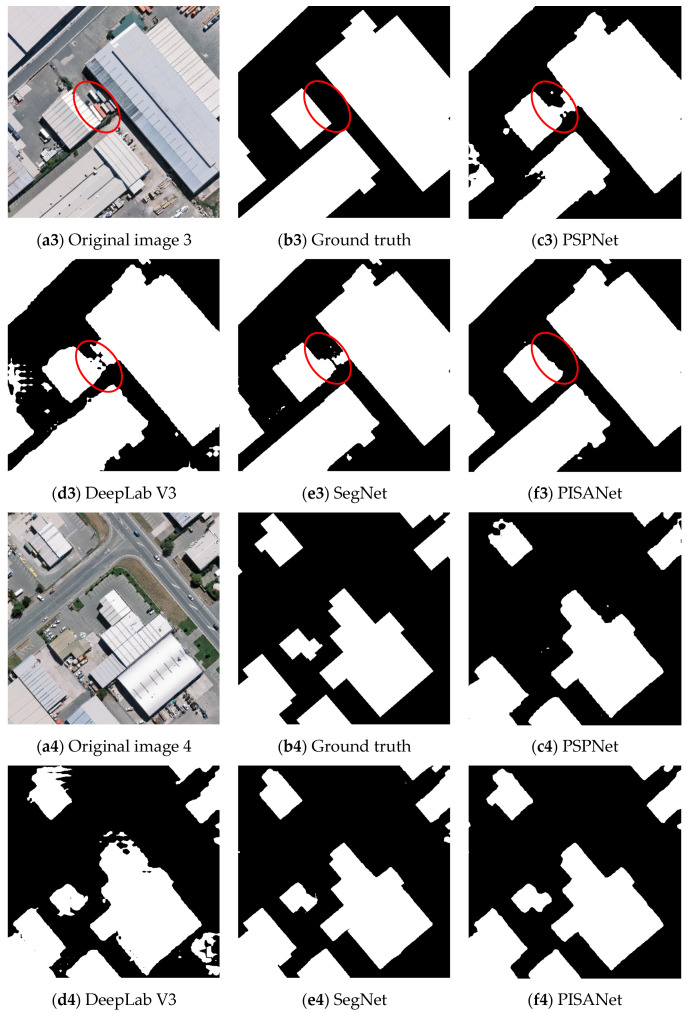
Visual comparison of various methods on WHU Building Dataset.

Some representative areas were selected to display the experimental results on the WHU Building Dataset for qualitative analysis (Figure 8). Original image 1 shows objects similar to containers, which are easily confused with buildings, original image 2 shows small buildings and original images 3 and 4 show large buildings with different shapes. Judging from the experimental results, PSPNet and DeepLab V3 were prone to leave buildings connected, did not distinguish individual buildings well and misclassified some of the non-building parts. SegNet and PISANet both performed well on large and small buildings. However, SegNet was more prone to incorrect extractions (red circle in Figure 8), and PISANet extracted buildings more accurately because it captures local and global features simultaneously.

The experimental results of PISANet and the comparison based on the WHU Building Dataset show that PISANet has the best performance for three important indicators OA, IoU and F1, which are, respectively, at least 0.48, 0.77 and 0.40% higher (Table 3).

**Figure 9:**
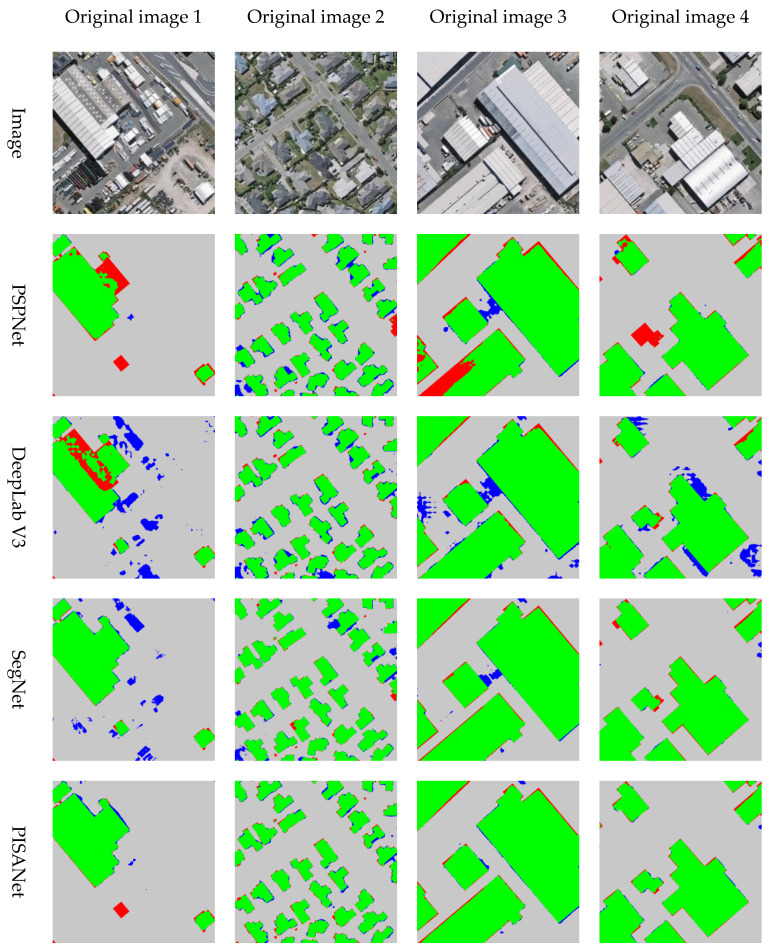
Visualization of various indicators of different methods on WHU Building Dataset, where gray is TN, green is TP, red is FN, blue is FP.

The extraction results were plotted in color on the WHU Building Dataset in Figure 9. From top to bottom, there is a significant reduction in the red and blue parts of the prediction results of each method in different regions. As shown in original image 1, PSPNet and DeepLab V3 show large red parts, indicating obvious omissions. DeepLab V3 and SegNet have large blue parts, indicating misclassification by these two methods.

## 5. Conclusions

In this paper, a Pyramid Self-Attention Network for building extraction from high spatial resolution remote sensing images was presented. Two public datasets, Inria Aerial Image Labeling Dataset and WHU Building Dataset, were used to verify the effectiveness of PISANet. These datasets contain buildings with highly diverse sizes, types and shapes. Experimental results have demonstrated that the PISANet made full use of the local and global information together and obtained higher final accuracy. Compared with other widespread networks, such as SegNet, PSPNet and DeepLab V3, the proposed PISANet can distinguish easily confused building-like objects, and the buildings that were partially obscured by shadows were accurately extracted and single buildings maintained better boundary integrity. PISANet has a simple model structure and only consists of a backbone and a pyramid self-attention module. Theoretically, the backbone can be replaced with any other network such as VGG, ResNet and ResNeXt. PISANet is suitable for extracting buildings from high spatial resolution remote sensing images, with simple structure and easy implementation.

## Figures and Tables

**Table 1:** Characteristics of Inria Aerial Image Labeling Dataset and WHU Building Dataset.

Dataset	Resolution	Image Size	Spectral	Image Counts	File Size	Cities	Area
Inria Aerial Image Labeling Dataset	0.3 m	5000 × 5000	Red/Green/Blue	360	12.7 G	Austin, Bellingham, Bloomington, Chicago, Innsbruck, Kitsap, San Francisco, Western and Eastern Tyrol, Vienna	810 km^2^
WHU Building Dataset	0.075~0.3 m	512 × 512	Red/Green/Blue	8188	6.1 G	Christchurch in New Zealand	450 km^2^

**Table 2:** Experimental results on the Inria Aerial Image Labeling Dataset.

Models	OA	IoU	Precision	Recall	F1
PSPNet	90.11	61.47	76.73	77.72	75.85
DeepLab V3	91.18	60.30	92.37	63.60	74.84
SegNet	94.24	76.71	85.04	88.71	86.80
PISANet	94.50	77.45	85.92	88.68	87.27

**Table 3:** Experimental results on the WHU Building Dataset.

Models	OA	IoU	Precision	Recall	F1
PSPNet	92.59	71.75	91.20	76.80	81.16
DeepLab V3	92.21	79.19	84.46	92.48	88.16
SegNet	95.67	87.20	93.29	93.05	93.05
PISANet	96.15	87.97	94.20	92.94	93.55
